# Association Between Vitamin D and Influenza: Meta-Analysis and Systematic Review of Randomized Controlled Trials

**DOI:** 10.3389/fnut.2021.799709

**Published:** 2022-01-07

**Authors:** Zhixin Zhu, Xiaoxia Zhu, Lanfang Gu, Yancen Zhan, Liang Chen, Xiuyang Li

**Affiliations:** Department of Epidemiology and Biostatistics, Center for Clinical Big Data and Statistics, Second Affiliated Hospital, College of Medicine, Zhejiang University, Hangzhou, China

**Keywords:** vitamin D supplementation, influenza, influenza-like illness, respiratory tract infection, meta-analysis

## Abstract

**Background:** Vitamin D supplementation improves the immune function of human body and can be a convenient way to prevent influenza. However, evidence on the protective effect of vitamin D supplementation on influenza from Randomized Controlled Trials (RCTs) is inconclusive.

**Methods:** RCTs regarding the association between vitamin D supplementation and influenza were identified by searching PubMed, Cochrane library, Embase and Chinese Biomedical Database (CBM) from inception until present (last updated on 10 November 2021). Studies that reported dosages and durations of vitamin D supplementation and number of influenza infections could be included. Heterogeneity was assessed using Cochran's Q test and *I*^2^ statistics, the meta-analysis was conducted by using a random-effects model, the pooled effects were expressed with risk ratio (RR) with 95% confidence interval (95% CI).

**Results:** 10 trials including 4859 individuals were ultimately eligible after scanning. There was no evidence of a significant heterogeneity among studies (*I*^2^ = 27%, *P* = 0.150). Meta-regression analysis finding indicated that country, latitude, average age, economic level, follow-up period and average daily vitamin D intake did not cause the statistical heterogeneity. The study finding indicates that substitution with vitamin D significantly reduces the risk of influenza infections (*RR* = 0.78, 95% CI:0.64–0.95). No evidence of publication bias was observed. Omission of any single trial had little impact on the pooled risk estimates.

**Conclusions:** The meta-analysis produced a corroboration that vitamin D supplement has a preventive effect on influenza. Strategies for preventing influenza can be optimized by vitamin D supplementation.

## Introduction

Influenza (referred to as flu) is an acute upper respiratory tract infection caused by influenza viruses. According to WHO, seasonal influenza epidemics cause an estimated 3 to 5 million severe cases and 290,000 to 650,000 deaths globally each year, indicating the substantial burden in public health worldwide with the impact of influenza (1). Although influenza vaccines are available, their efficacy is declined in mismatched seasons (2).

There are several studies concerning the effect of vitamin D supplementation on the prevention of influenza infection have been reported, but their results were inconsistent. Zhou et al. preformed an RCT in infants and found a significant difference between low-dose and high-dose vitamin D groups in the occurrence of influenza an infection (*RR* = 0.56, 95% CI: 0.42–0.77) (3). Aglipay et al. also reported a significant difference (*RR* = 0.50, 95% CI: 0.28–0.89) (4). Additionally, Urashima et al. conducted a RCT in 2010, and the result showed that vitamin D3 supplementation during the winter reduced the incidence of influenza A (*RR* = 0.58, 95% CI: 0.34–0.99) (5). In 2014, Urashima et al. preformed another RCT and reported that during the first month of follow-up, vitamin D3 supplementation was a protective factor against influenza (*RR* = 1.11, 95% CI: 0.57–2.18), but based on the observation results at two-month follow-up, vitamin D supplementation does not play a role in preventing influenza (*RR* = 0.17, 95% CI:0.04–0.77) (6), which indicated that only short-term use of vitamin D3 dietary supplementation can temporarily decrease the incidence of influenza A during an influenza pandemic. However, Arihiro et al. have observed an insignificant difference in influenza incidence between the vitamin D and placebo group (*RR* = 1.25, 95%CI: 0.45–3.49) (7). Loeb et al. also reported ineffectiveness of vitamin D supplementation in preventing influenza (8). Studies found that vitamin D deficiencies in blood was associated with the increased incidence of respiratory tract infections and influenza (9–13). However, some opposite results were reported (14, 15).

In order to determine the overall effect of vitamin D supplementation on the risk of influenza and to identify factors that might influence the effects of this intervention on the risk of influenza, a meta-analysis and system review of RCTs was conducted in the study.

## Materials and Methods

### Search Strategy

This systematic review and meta-analysis were conducted according to the Cochrane Handbook for Systematic Reviews of Interventions and the Preferred Reporting Items for Systematic Reviews and Meta-Analysis guidelines for meta-analysis (16, 17). Randomized Controlled Trials (RCTs) and clinical trials published before 10 November 2021 were collected from the electronic databases such as PubMed, Cochrane Library, Embase and CBM. Additionally, the OpenGrey database (http://www.opengrey.eu/) was also searched for any potential studies, and the International Clinical Trials Registry Platform (http://apps.who.int/trialsearch/) was scanned for ongoing trials. The references of retrieved studies and reviews were manually crosschecked.

Research terms “vitamin D or ergocalciferols or cholecalciferol” AND “influenza or pneumonia or common cold or respiratory tract infection or influenza-like illness or ILI” were used. The details of research strategy were provided in Supplementary Table S1. Each identified report was carefully scanned by two of the authors.

### Selection Criteria

The inclusion criteria were as follows: (1) RCTs exploring the effect of vitamin D supplementation on the prevention of influenza; (2) studies reporting the dosages, modes and duration of vitamin D supplementation; (4) studies reporting the definitions of influenza patients or ILI (influenza-like illness) and the methods used to diagnose them. (5) English or Chinese articles. Reports as follow were excluded: (1) case reports, reviews, letters or comments; (2) animal or cell trials; (3) not involved in the association between vitamin D and influenza patients or ILI.

### Data Collection and Quality Assessment

Two reviewers used Endnote9x software to screen literature. Data information were extracted through a standardized data extraction form. If there was any disagreement among two reviewers, the report would be sent to a third researcher and fully discussed. The Cochrane Collaboration's tool for assessing risk bias were used to assess the experimental study's quality, based on six quality criteria: sequence generation, allocation concealment, blinding; missing outcome data, selective reporting and other biases (18). Review Manager (version 5.4.1) was used here. The overall confidence in the estimate was assessed with GRADE profiler software according to the criteria published by the GRADE working group (19). The extracted information from included reports consisted of first author's name, publication year, participants, sample size, location, average age, dose design, follow-up period, season and primary outcomes.

### Primary Outcomes

The primary outcome measures of the present study were the prevention of influenza infections in human including laboratory-confirmed influenza infections and ILI (body temperature greater than or equal to 38°C, accompanied by either cough or pharyngeal pain, while other laboratory diagnostic evidence is lacking).

### Statistical Analysis

Study results were quantitatively combined for each outcome. For dichotomous outcomes, pooled estimates expressed as relative risks (RR) with the corresponding 95% confidence intervals (95%CIs) were calculated. *Q* test and I^2^-statistic with its 95%CI were used to assess the heterogeneity among the effect results (20, 21), and the pooled RR was computed using a random-effects model because of the different characteristics of included studies such as diverse populations and different background diseases of subjects. If *P*-value of the *Q* test is greater than 0.05 and *I*^2^ ≤ 50%, we can consider that there was no statistically heterogeneity. Forest plots were produced to show each trial's result and estimate the pooled effect sizes. Publication bias was tested by funnel plots, using Harbord tests (22). Sensitivity analysis was used to measure the impact of each individual trial on the combined effects and the robustness of results.

Statistical analysis was performed with Review Manager (version 5.4.1) and STATA 14.0 software. Meta-regression analysis was conducted to verify whether some covariates such as country, latitude (<30, 30–50 or >50°), average age (18<, 18–50 or >50 years), economic level (developed or developing), average daily vitamin D intake (<2000 or ≥2000 IU/day) and follow-up period (<4, 4–8 or ≥ 8 months) would influence the association between vitamin D intake and influenza, and we could confirm the influence factor with a positive coefficient (*P* ≤ 0.05). We also performed two subgroup analyses (adult vs. children and winter vs. all seasons) to explore in depth the respective influence of developmental stage and season on the effect. Some of the age data were mathematically processed (23, 24).

## Results

### Search Results and Trial Characteristics

**Figure 1:**
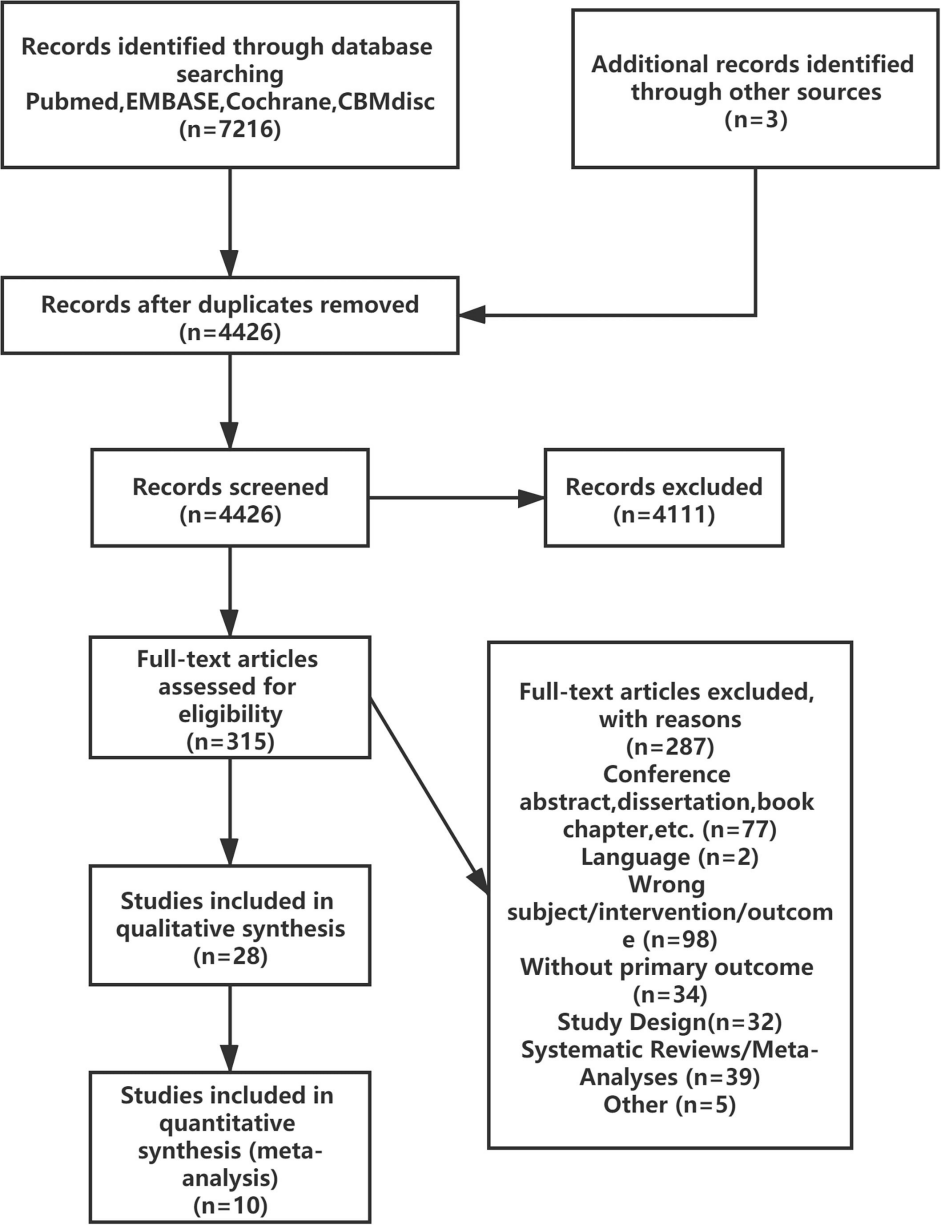
PRISMA flow diagram of the study selection process.

The literature screening process is presented in Figure 1; 7216 reports were identified after the databases were searched, and 28 reports were retained after reviewing the title and summary according to the exclusion criteria. Then 18 reports were excluded after full text review. A total of 10 RCTs were satisfied the criteria for inclusion and entered the meta-analysis (3–8, 25–28).

**Figure 2:**
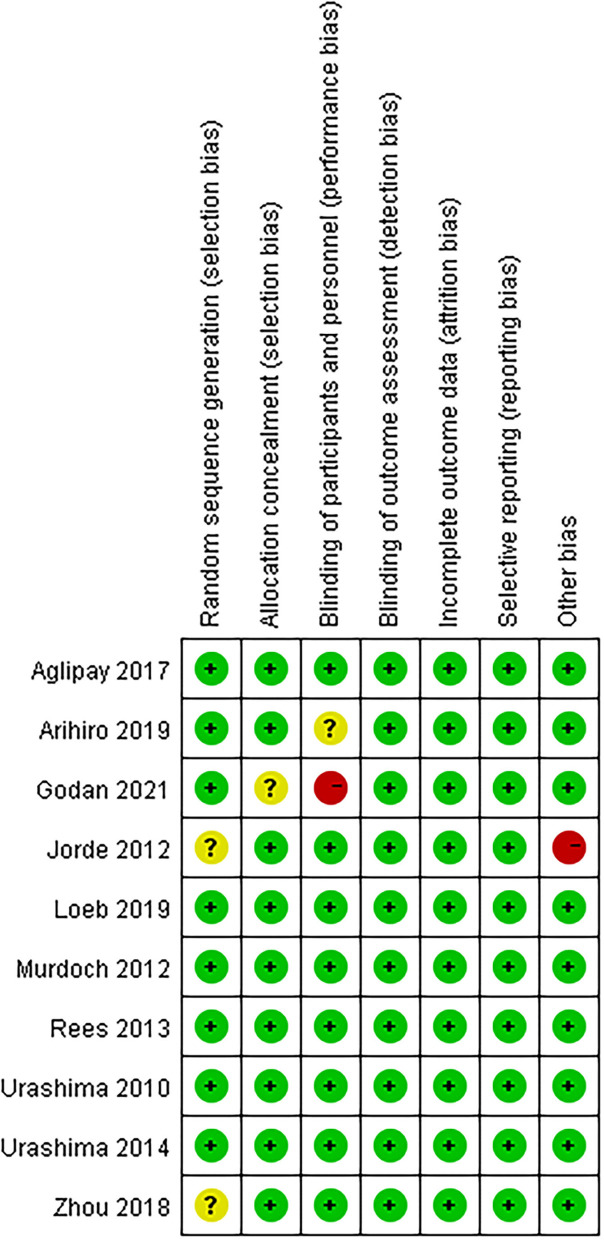
Risk of bias summary: review authors' judgements about each risk of bias item for each included study.

Details of the study characteristics were shown in Table 1. The included studies were mainly conducted in Asian, North America or Europe countries, and were all published after the influenza epidemic in 2009. All used oral cholecalciferol of these studies, six used laboratory examinations like Polymerase Chain Reaction and two used a questionnaire to diagnosis influenza, and one contained both methods. Most of the participants were healthy adults, and the number of them was almost the same for men and women (4026:4003). The risk of bias was assessed using a risk-of-bias graph (Figure 2). Most of the RCT exhibited good allocation concealment, reporting of blinding methods, and complete outcome data; however, one study had a high risk in blinding of participants (25), and the other had a high risk of other biases due to its procedure and diagnosis method (27). Due to the inconsistency of studies in the primary outcomes, we therefore downgraded the overall quality of evidence (Supplementary Table S2).

**Table 1:** Characteristics of clinical trials included in the meta-analysis.

**1st author**	**Year**	**Location**	**Latitude**	**Economic level**	**Participants**			**Dosing regime**		**Follow–up period**	
					**Males/Females**	**Average age (range)**	**Health status**	**Vitamin D group**	**Control**	**Duration**	**Season**
Aglipay et al. (4)	2017	Toronto, Canada	43°N	Developed	404/296	2.7 (1–5) years	Healthy children	2000 IU/Day	400IU/Day	8 months	Winter
Arihiro et al. (7)	2019	Tokyo, Japan	35°N	Developed	136/87	44 (18–50) years	Adults with ulcerative colitis or Crohn's disease	500 IU/Day	Placebo	6 months	Winter
Loeb et al. (8)	2019	Ha Nam Province, Vietnam	21°N	Developing	621/679	8.5 (3–17) years	Healthy children and teenagers	2000 IU/Day	Placebo	12 months	All seasons
Urashima et al. (5)	2010	Tokyo, Japan	35°N	Developed	242/188	10.2 (6–15) years	Healthy children and teenagers	1200 IU/Day	Placebo	4 months	Winter
Urashima et al. (6)	2014	Tokyo, Japan	35°N	Developed	162/83	(15–18) years	Healthy teenagers	2000 IU/Day	Placebo	1 month	Winter
Zhou et al. (3)	2018	Yongkang, China	29°N	Developing	173/159	8 (3–12) months	Healthy infants	1200 IU/Day	400IU/Day	8 months	Winter
Godan et al. (25)	2021	Zagreb, Croatia	46°N	Developing	26/71	78.5 (61–96) years	Elderly patients with different kinds of chronic disease	800 IU/Day	Blank control	3 months	Winter
Rees et al. (28)	2013	Houston, Atlanta et.al	30–36°N	Developed	438/321	57.8 (45–75) years	Adults with a history of colorectal adenoma	1000 IU/Day	Placebo	5 months	Winter
Murdoch et al. (26)	2012	Christchurch, New Zealand	43°S	Developed	81/241	47 years	Healthy adults	200,000 IU/monthly for 2 months; 100,000 IU/monthly	Placebo	18 months	All seasons
Jorde et al. (27)	2012				327/242	63 (32–84) years					
Jorde(1)		Troms, Norway	69°N	Developed		63 (34–82) years	1.Subjects with reduced glucose tolerance 2.Healthy adults	20000–40000 IU/week	Placebo	3 months	Winter
Jorde(2)		Vienna, Austria	48°N	Developed		61 (32–72) years	Kidney transplant recipient	6800 IU/Day	Placebo	3 months	Winter
Jorde(3)		Seattle, USA	47°N	Developed		60 (46–76) years	Subjects with Type 2 diabetes	2000 IU/Day	Placebo	3 months	Winter
Jorde(4)		Dundee, Scotland	47°N	Developed		71 (50–84) years	1.Isolated systolic hypertension 2. Adults with a past history of myocardial infarction 3.Adults with resistant hypertension	100,000 IU/quarterly	Placebo	3 months	Winter
Jorde(5)		Aarhus, Denmark	56°N	Developed		65 (33–77) years	Healthy adults	2800 IU/Day	Placebo	3 months	Winter
Jorde(6)		Leuven, Belgium	51°N	Developed		72 (56–82) years	Patients with moderate to very severe COPD	100,000 IU/monthly	Placebo	3 months	Winter

### Effects of Vitamin D Supplementation on the Primary Outcomes

**Figure 3:**
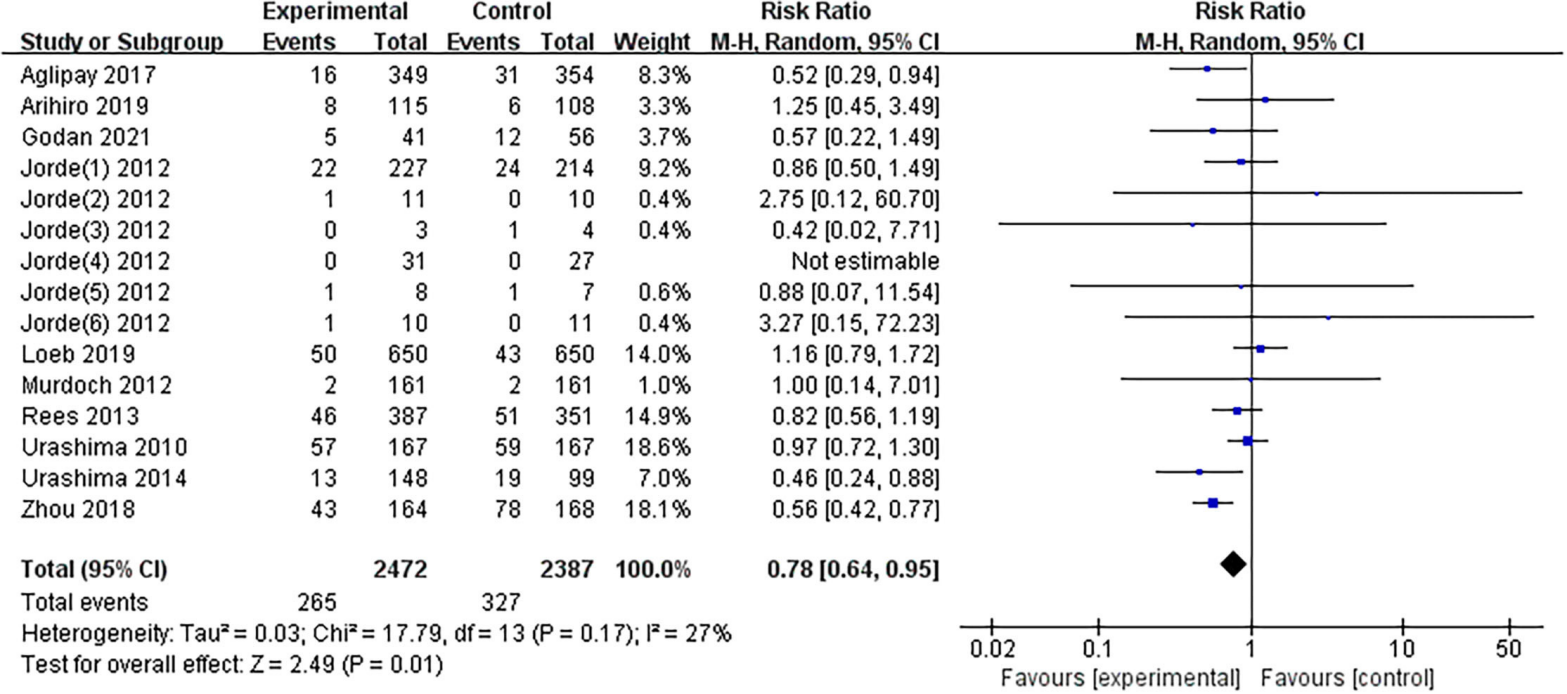
Forest plot of comparisons of vitamin D vs. control on influenza. M-H, Mantel-Haenzsel statistics; Random, Random effects model.

The results of one article (27) were divided into six parts by region. The results of the overall meta-analysis were presented in Figure 3. The summarized results of the included studies indicated that substitution with vitamin D significantly reduces the risk of influenza infections (*RR* = 0.78, 95% CI: 0.64–0.95). There was no evidence of a significant heterogeneity among studies (*I*^2^ = 27%, *P* = 0.170). The 95% CI for *I*^2^ was 0–61%.

Meta-regression analysis was conducted to verify the possible influential factors, and the result showed in Table 2 manifested that country, latitude (<30, 30–50 or >50°), average age (18<, 18–50 or >50 years), economic level (developed or developing), average daily vitamin D intake (<2000 or ≥2000 IU/day) and follow-up period (<4, 4–8 or ≥ 8 months) were not influential factors. Therefore, all 14 estimates were incorporated into the meta-analysis.

**Table 2:** Characteristics of univariate meta–regression analysis.

**Covariates**	**Coefficient**	**SE**	***t* value**	***P* value**
Country	0.988	0.109	−0.110	0.917
Latitude	1.005	0.783	0.010	0.995
Economic level	0.959	0.785	−0.050	0.960
Age	1.190	0.412	0.500	0.631
Follow–up period	1.137	0.370	0.390	0.706
Average daily vitamin D intake	1.032	0.383	0.080	0.935
Knapp–Hartung modification				0.997

There was no significant difference in effect between children group and adults' group as was showed in Supplementary Figure S1 (*P* = 0.470). For season, the pooled *RR* of all seasons' group was 1.16 (95%CI: 0.79–1.70, *I*^2^ = 0%), while the pooled *RR* of winter group was 0.73 (95%CI: 0.60–0.88, *I*^2^ = 17%). Difference in heterogeneity between the two subgroups was statistically significant (*P* = 0.040), and vitamin D supplementation in winter might have a better effect of preventing influenza than in all seasons (Supplementary Figure S2).

### Publication Bias and Sensitivity Analyses

**Figure 4:**
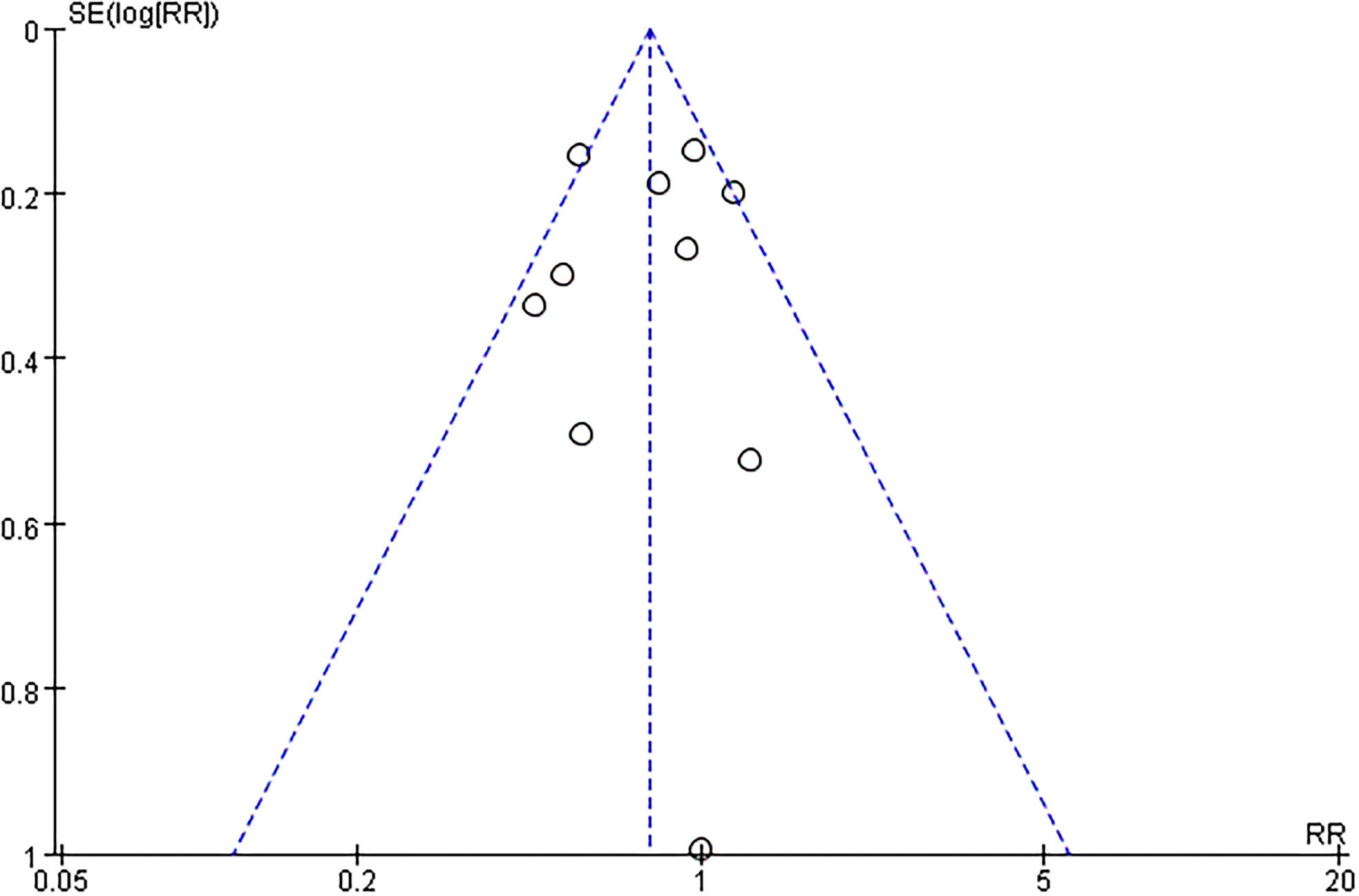
Funnel plots with pseudo 95% confidence intervals.

**Figure 5:**
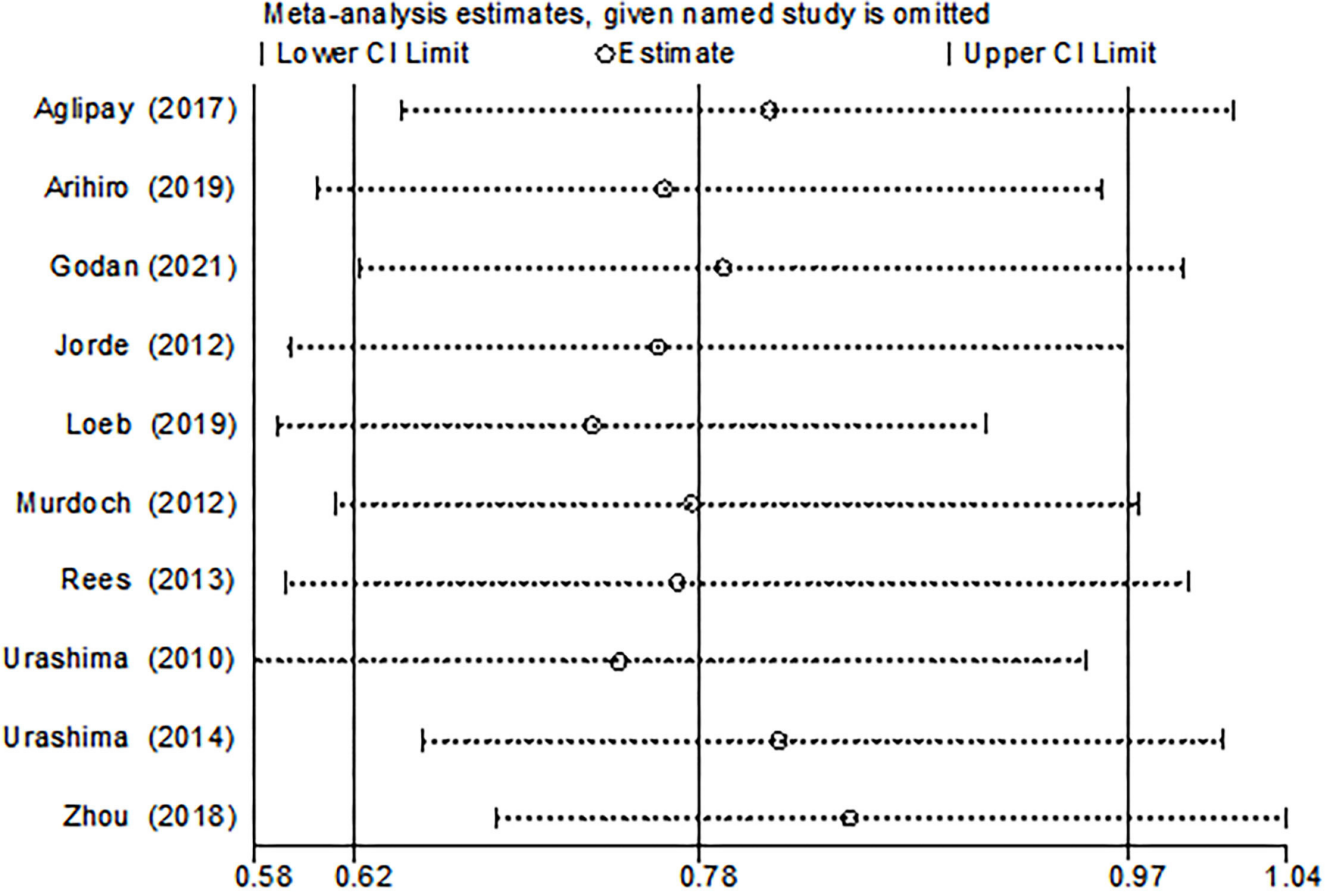
Results of sensitivity analysis.

The funnel plot, as shown in Figure 4, suggested we could rule out the publication bias (Harbord tests *t* = −0.08, *P* = 0.937). The meta-analysis result was robust according to the sensitivity analysis as shown in Figure 5 and Table 3. Each trial had a modest influence on the overall results, and after exclusion of single studies the estimated *RR* remained within the range 0.73(95%CI: 0.59–0.90) to 0.84(95%CI: 0.69–1.04).

**Table 3:** Sensitivity analysis results.

**Study omitted**	**Estimate**	**95%CI**
Aglipay et al. (4)	0.806	0.643–1.012
Arihiro et al. (7)	0.760	0.605–0.953
Godan et al. (25)	0.786	0.624–0.990
Jorde et al. (27)	0.757	0.593–0.966
Loeb et al. (8)	0.728	0.588–0.902
Murdoch et al. (6)	0.772	0.614–0.970
Rees et al. (28)	0.766	0.598–0.992
Urashima et al. (5)	0.740	0.578–0.947
Urashima et al. (6)	0.810	0.652–1.007
Zhou et al. (3)	0.842	0.685–1.035

## Discussion

Several mechanisms explain the possible preventive effects of vitamin D supplementation on influenza. A recent review grouped those mechanisms into three categories: physical barrier, cellular natural immunity, and adaptive immunity (29). Cells involved in innate and adaptive immunity contain the 1-alpha-hydroxylase in their mitochondria. This series of cells including macrophages, neutrophils, dendritic cells, natural killer cells, B cells, CD4 and CD8 cells also contain surface Vitamin D Receptors (30). Calcitriol regulates local immune function by binding to Vitamin D Receptors through endocrine, paracrine, and autocrine mechanisms that influence gene transcription. Calcitriol also has a broader role in regulating secondary expression of some genes responsible for transcription of pro-inflammatory cytokines (31).

This meta-analysis of 10 RCTs indicates a protective effect of vitamin D supplementation against influenza infections with a combined risk ratio of 0.78 (95% CI: 0.64–0.95; *P* = 0.010). It is difficult to compare this study with the previous analysis because no one has done the same analysis before. Some previous meta-analyses studied the effect of vitamin D supplementation against respiratory tract infections and got inconsistent results (32–36), two of which with no restriction imposed on the origin of participants are consistent with our conclusion (32, 36), but none of them analyzed influenza as a subgroup.

Many factors such as region, age, economic level, follow-up period and average daily vitamin D intake may affect the serum vitamin D level of subjects or the mode of action of vitamin D supplementation, and then influence the outcome. Thus, we took these factors into meta-regression analysis but showed no marked impact on the conclusions.

Regions have an impact on vitamin D status and influenza infections. Different people in countries with different levels of economic development are various in vitamin D intake. Influenza mainly occurs in winter, so we mainly extracted outcome data from winter episodes (28). Winter influenza peak was due in part to the conjunction with the season when solar UVB doses, and thus 25(OH)D concentrations, are lowest in most mid- and high-latitude countries (37). Therefore, it seems reasonable that vitamin D supplementation is more effective in preventing influenza during the winter. However, Rees et al. reported null effects both in winter and all seasons (28). Most of the other included studies were conducted in winter, and only two reported the effectiveness (3, 4). The amount of ultraviolet radiation b (UVB) decreases along latitude in winter, and the UVB-related vitamin D production decreases dramatically in the areas at latitudes above 40° (38). But here, latitudes seem to have no influence on the effect of vitamin D.

Infants may lack sun exposure because they are more likely to stay at home, which causes reduced cutaneous synthesis of vitamin D3. Serum 25(OH)D concentrations tend to decline with age because aging is associated with decreased levels of 7-dehydrocholesterol, the precursor of vitamin D3 in the skin (39, 40). And both the old and infants have weak immunity and are more susceptible to influenza virus. We suspect that the preventive effect of vitamin D on influenza is more obvious in infants and the elderly. 2 studies reported the vitamin D was effective in preventing influenza in infants, which was in line with prat of our conjectures (3, 4). However, the results of meta regression and subgroup analysis (children vs. adults) showed that there were not any significant differences in effects between different age groups. In teenagers and the elderly, 5 studies included here demonstrated a null effect (5, 6, 8, 27, 28). This may be because the subjects in these studies had higher baseline serum vitamin D levels, which compromised the effect of vitamin D supplement. Arihiro et al. reported 58.7 nmol/l (7); Loeb et al. reported 65nmol/l (8); Rees et al. reported 73.4 nmol/l (28), and Murdoch et al. reported 72.3 nmol/l (26). In a cohort study, participants with predicted baseline serum 25(OH)D ≥ 50 nmol/L had lower risk in influenza infection compared with 25(OH)D ≤ 50 nmol/L group after analyzing the combined outcome of PCR-confirmed influenza virus infection and ILI (*RR* = 0.63, 95% CI:0.52–0.76) (41). Given the higher serum vitamin D levels in our included observation subjects, as well as the lower incidence in the high vitamin D level population, differences in effects between the intervention and control groups are difficult to detect.

Vitamin D metabolites have different effects on the immune response of various respiratory viruses and regulate the secretion of cytokines (42). The cytokine secretion pattern are different between influenza A and influenza B (43). So preventive effects of vitamin D on influenza A and influenza B may also be different. Urashima et al. reported that vitamin D reduced the incidence of influenza A (*RR* = 0.58, 95% CI:0.34–0.99), but not of influenza B (*RR*= 1.39, 95% CI:0.90–2.15) (5). We estimated RR value for influenza A (*RR*= 0.36, 95% CI: 0.15–0.85) and influenza B (*RR* = 0.83, 95% CI: 0.04–18.00) respectively based on Aglipay's research outcomes by using Woolf's method, and the conclusion was consistent with Urashima's (4). But in Loeb's study, vitamin D supplement had no effect on both influenza A and B (8). This difference may be due to the experimental design and some biases, we still assume that vitamin D supplement is likely to have better effects on preventing influenza A than B.

This meta-analysis produced a corroboration that vitamin D supplement has a preventive effect on influenza, and vitamin D supplementation in winter seems to have better effect of preventing influenza than in all seasons according to subgroup analysis (winter vs. all seasons). Compared with drugs and influenza vaccines, vitamin D supplementation as a way to prevent influenza is more convenient and more acceptable due to its safety and many other benefits in maintaining bone health. Vitamin D did not have an impact on immunological effects of influenza vaccines (44, 45). So, it is recommended that people of all age can supplement vitamin D to prevent influenza and get vaccinated if necessary, during wintertime. Because of the small number of included reports and incomplete reporting of serum vitamin D levels, we did not further analyze the appropriate doses of vitamin D supplementation in different age groups. However, vitamin D supplementation may have different effects on people of various age levels, which is closely related to the physiological condition of the patients themselves. So, researchers should develop different vitamin D supplementation programs for them. Our findings can help optimize the influenza prevention strategies and provide a theoretical basis for the development of nutrition guidelines.

Our study has several strengths. First, all the enrolled studies were RCTs, which eliminates the possibility of reverse causation and minimizes selection and recall biases, while the non-RCT design has more of the above biases and confounding factors. Second, we have conducted relative comprehensive analyses, including meta-regression analysis, influence analysis and publication bias detection, to explore potential sources of heterogeneity and bias. Furthermore, no restriction was imposed on the origin of participants, which may generalize the results.

The limitation of this meta-analysis cannot be ignored: First, four articles did not report baseline serum vitamin D levels, and therefore, we could not analyze whether the baseline serum concentrations of 25(OH)D modified the effects of vitamin D supplementation. Second, two study with a relative high risk of bias was included in the analysis. Fortunately, the exclusion of these studies had only modest influence on the outcome based on the results of sensitivity analysis. Finally, the age range of the subjects in this study is very wide (3 months to 82 years). Due to the small number of studies, the three categories used in this study alone may not show a possible difference among subgroups.

## Conclusions

Aggregated evidence from 10 RCTs indicates that vitamin D supplementation could be an effective for preventing influenza. This meta-analysis was conducted based on RCTs, so the strength of evidence argument is very strong. Our findings can provide a theoretical basis for the development of nutrition guidelines.

## Data Availability Statement

The original contributions presented in the study are included in the article/Supplementary Material, further inquiries can be directed to the corresponding author/s.

## Author Contributions

XL and ZZ conceived and designed the meta-analysis. ZZ, XZ, LG, and YZ searched the literature. ZZ and XZ extracted the data. ZZ and LC analyzed the data. ZZ contributed analysis tools and wrote the paper. XL revised the manuscript. All authors contributed to the article and approved the submitted version.

## Funding

This work was supported by the Soft Science Key Project of the Science and Technology Department of Zhejiang Province (2019C25009 and 2022C25040), the Key Project of Social Science Planning in Hangzhou City (hzjz20180110), and the Soft Science Key Project of Hangzhou Municipal Science Committee (20160834M03).

## Conflict of Interest

The authors declare that the research was conducted in the absence of any commercial or financial relationships that could be construed as a potential conflict of interest.

## Publisher's Note

All claims expressed in this article are solely those of the authors and do not necessarily represent those of their affiliated organizations, or those of the publisher, the editors and the reviewers. Any product that may be evaluated in this article, or claim that may be made by its manufacturer, is not guaranteed or endorsed by the publisher.

## Supplementary Material

Supplementary Figure S1 Forest plot of subgroup analysis (adults vs. children).

Supplementary Figure S2 Forest plot of subgroup analysis (winter vs. all seasons).

Supplementary Table S1 Search strategy and search terms.

Supplementary Table S2 GRADE level of evidence and summary of findings of vitamin D for the prevention of influenza.
