# Supplementary figures and images for: Association Between Vitamin D and Influenza: Meta-Analysis and Systematic Review of Randomized Controlled Trials
Source: Front Nutr. 2022 Jan 7;8:799709. doi: 10.3389/fnut.2021.799709 (PMC8777486; doi:10.3389/fnut.2021.799709)

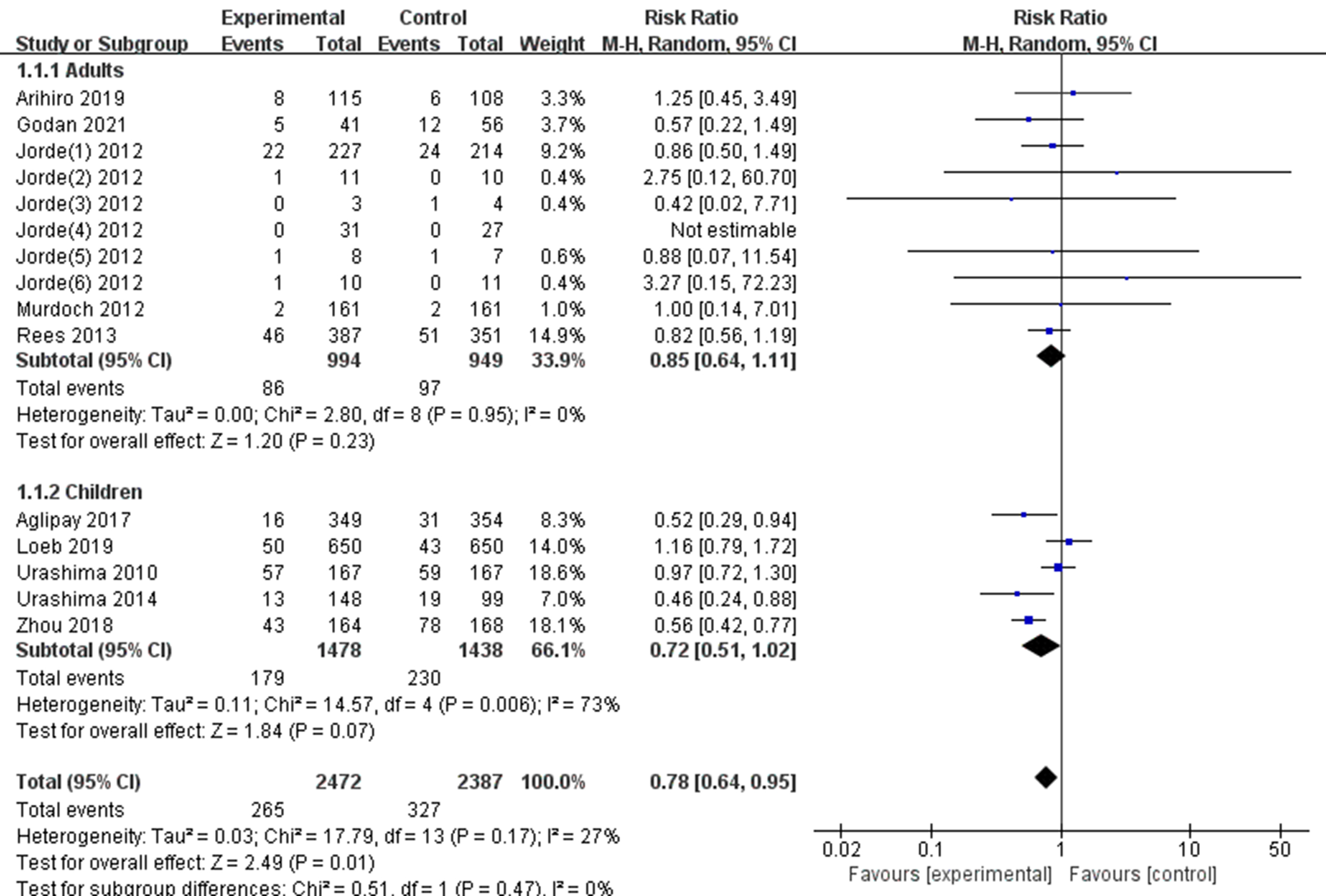

Supplement: Supplementary Figure S1 — Forest plot of subgroup analysis (adults vs. children). [file Image_1.JPG]

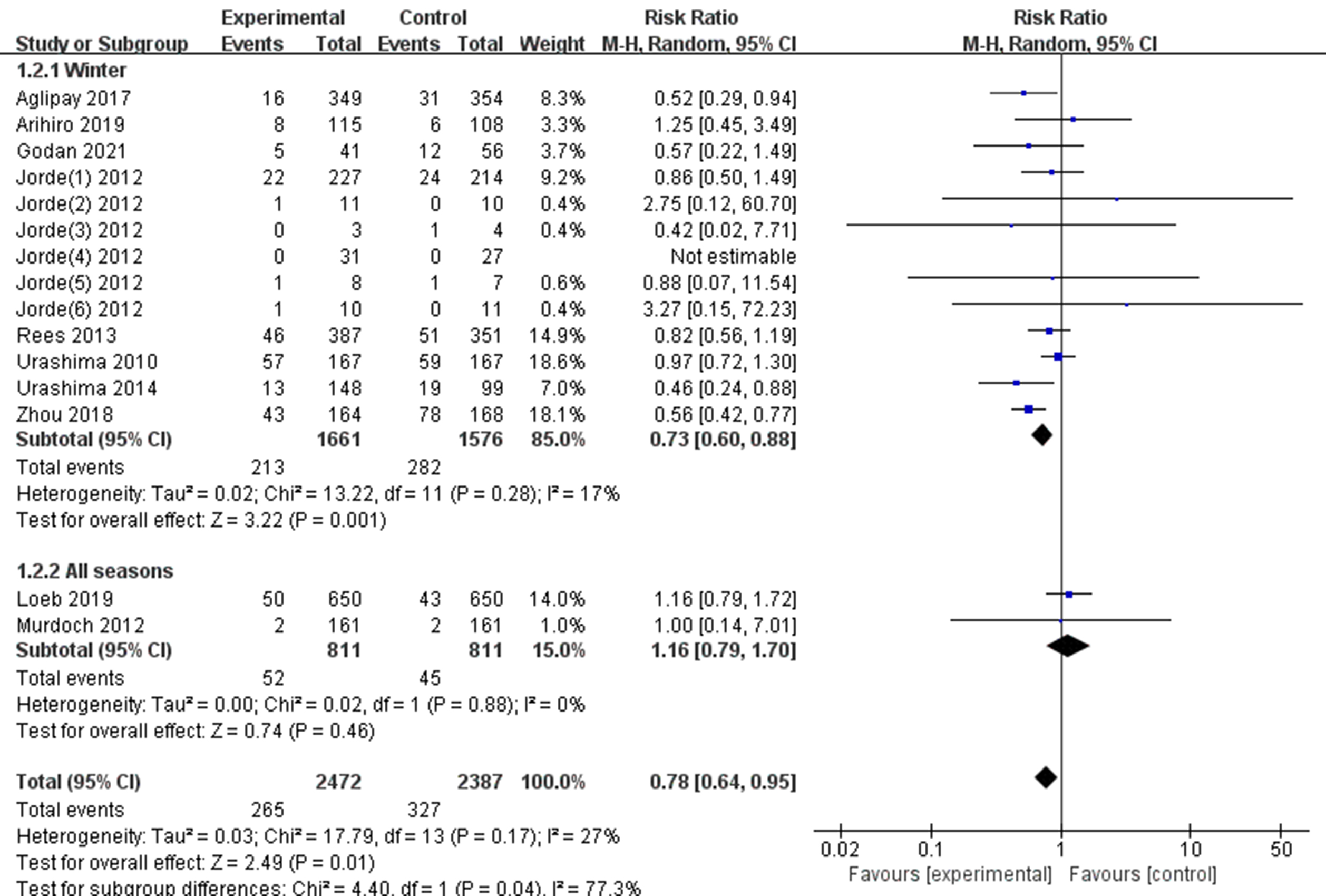

Supplement: Supplementary Figure S2 — Forest plot of subgroup analysis (winter vs. all seasons). [file Image_2.JPG]
