# Supplementary material for: Association Between Vitamin D and Influenza: Meta-Analysis and Systematic Review of Randomized Controlled Trials
Source: Front Nutr. 2022 Jan 7;8:799709. doi: 10.3389/fnut.2021.799709 (PMC8777486; doi:10.3389/fnut.2021.799709)
Supplement: Supplementary Table S1 — Search strategy and search terms. [file Table_1.docx]

**Table S1. Search strategy and search terms**

| No. | Search Terms | | | | Number of Articles* | |  |
| --- | --- | --- | --- | --- | --- | --- | --- |
| Pubmed | | | | | | |  |
| #1 | [MeSH Term] | | Vitamin D | | 63698 | |  |
| #2 | [Text Word] | | vitamin D or vitamin D2 or vitamin D3 or | | 84147 | |  |
| #3 | [Text Word] | | al*acalcidol or c?olecalciferol or calcitriol or calcidiol or calcifediol or calciferol or calciol or calderol or dihydrotachysterol or dedrogyl or dihydrotachysterol or dihydroxycolecalciferol or dihydroxycholecalciferol or dihydroxyvitamin D or dihydroxyvitamin D2 or dihydroxyvitamin D3 or doxercalciferol or eldecalcitol or ercalcidiol or ergocalciferol* or hidroferol or hydroxycalciferol or hydroxyl‐calciferol or hydroxycolecalciferol or hydroxycholecalciferol or hydroxyergocalciferol* or hydroxyvitamin D or hydroxyvitamin D2 or hydroxyvitamin D3 or paricalcitol or tachystin | | 52127 | |  |
| #4 |  | | #1 OR #2 OR #3 | | 98547 | |  |
| #5 | [MeSH Term] | | Influenza, Human | | 53381 | |  |
| #6 | [MeSH Term] | | Respiratory Tract Infections | | 489406 | |  |
| #7 | [MeSH Term] | | Pneumonia | | 215523 | |  |
| #8 |  | | #4 OR #5 OR #6 | | 489406 | |  |
| #9 | [Text Word] | | respiratory tract infections or pneumonia or flu or flu like syndrom* or flu like symptom* or influenza like illness* or bronchit* or bronchopneumonia | | 298553 | |  |
| #10 |  | | #7 OR #8 | | 580644 | |  |
| #11 |  | | #4 AND #10 | | 1787 | |  |
| Cochrane Library | | | | | | | |
| #1 | | [MeSH] Explode all trees | | Vitamin D | | 5807 | |
| #2 | | [All Text] | | vitamin D or vitamin D2 or vitamin D3 | | 18678 | |
| #3 | | [All Text] | | al*acalcidol or c?olecalciferol or calcitriol or calcidiol or calcifediol or calciferol or calciol or calderol or dihydrotachysterol or dedrogyl or dihydrotachysterol or dihydroxycolecalciferol or dihydroxycholecalciferol or dihydroxyvitamin D or dihydroxyvitamin D2 or dihydroxyvitamin D3 or doxercalciferol or eldecalcitol or ercalcidiol or ergocalciferol* or hidroferol or hydroxycalciferol or hydroxyl‐calciferol or hydroxycolecalciferol or hydroxycholecalciferol or hydroxyergocalciferol* or hydroxyvitamin D or hydroxyvitamin D2 or hydroxyvitamin D3 or paricalcitol or tachystin | | 8740 | |
| #4 | |  | | #1 OR #2 OR #3 | | 20528 | |
| #5 | | [MeSH] Explode all trees | | Influenza,human | | 2824 | |
| #6 | | [MeSH] Explode all trees | | Pneumonia | | 4688 | |
| #7 | | [MeSH] Explode all trees | | Respiratory Tract Infections | | 16475 | |
| #8 | |  | | #5 OR #6 OR #7 | | 16475 | |
| #9 | | [All Text] | | respiratory tract infections or pneumonia or flu or flu like syndrom* or flu like symptom* or influenza like illness* or bronchit* or bronchopneumonia | | 30996 | |
| #10 | |  | | #8 OR #9 | | 39170 | |
| #11 | |  | | #4 AND #10 | | 733 | |
| Embase | | | | | | | |
| #1 | | [Emtree trem] Explode all trees | | vitamin D | | 161122 | |
| #2 | | keywords | | vitamin D or vitamin D2 or vitamin D3 | | 29457 | |
| #3 | | keywords | | al*acalcidol or c?olecalciferol or calcitriol or calcidiol or calcifediol or calciferol or calciol or calderol or dihydrotachysterol or dedrogyl or dihydrotachysterol or dihydroxycolecalciferol or dihydroxycholecalciferol or dihydroxyvitamin D or dihydroxyvitamin D2 or dihydroxyvitamin D3 or doxercalciferol or eldecalcitol or ercalcidiol or ergocalciferol* or hidroferol or hydroxycalciferol or hydroxycolecalciferol or hydroxycholecalciferol or hydroxyergocalciferol* or hydroxyvitamin D or hydroxyvitamin D2 or hydroxyvitamin D3 or paricalcitol or tachystin | | 77739 | |
| #4 | |  | | #1 OR #2 OR #3 | | 164365 | |
| #5 | | [Emtree trem] Explode all trees | | Influenza | | 102839 | |
| #6 | | [Emtree trem] Explode all trees | | Pneumonia | | 361487 | |
| #7 | | [Emtree trem] Explode all trees | | Respiratory Tract Infections | | 509047 | |
| #8 | |  | | #5 OR #6 OR #7 | | 725677 | |
| #9 | | keywords | | respiratory tract infections or pneumonia or flu or flu like syndrom* or flu like symptom* or influenza like illness* or bronchit* or bronchopneumonia | | 38702 | |
| #10 | |  | | #8 OR #9 | | 733127 | |
| #11 | |  | | #4 AND #10 | | 3975 | |

*China Biomedical Database(CBM) was also searched and 721 articles were found
