# Supplementary material for: Association Between Vitamin D and Influenza: Meta-Analysis and Systematic Review of Randomized Controlled Trials
Source: Front Nutr. 2022 Jan 7;8:799709. doi: 10.3389/fnut.2021.799709 (PMC8777486; doi:10.3389/fnut.2021.799709)
Supplement: Supplementary Table S2 — GRADE level of evidence and summary of findings of vitamin D for the prevention of influenza. [file Table_2.docx]

| **Supplementary Table S2. GRADE level of evidence and summary of findings of vitamin D for the prevention of influenza.** | | | | | | | | | | | |
| --- | --- | --- | --- | --- | --- | --- | --- | --- | --- | --- | --- |
| **Certainty assessment** | | | | | | | **Summary of findings** | | | | |
| **Participants (studies) Follow-up** | **Risk of bias** | **Inconsistency** | **Indirectness** | **Imprecision** | **Publication bias** | **Overall certainty of evidence** | **Study event rates (%)** | | **Relative effect (95% CI)** | **Anticipated absolute effects** | |
|  |  |  |  |  |  |  | **With placebo** | **With vitamin D supplementation** |  | **Risk with placebo** | **Risk difference with vitamin D supplementation** |
| **Influenza infections** | | | | | | | | | | | |
| 4859 (10 RCTs) | not serious | serious^a^ | not serious | not serious | none | ⊕⊕⊕⊝  Moderate | 327/2387 (13.7%) | 265/2472 (10.7%) | **RR 0.78** (0.64 to 0.95) | 137 per 1,000 | **30 fewer per 1,000** (from 49 fewer to 7 fewer) |

**CI:** confidence interval; **RR:** risk ratio

#### Explanations

a. Only 3 studies reported significant difference while 7 reported no significant difference
